# Intra-individual variance of bilateral femoro-tibial leg rotation: a CT study of 105 healthy subjects

**DOI:** 10.1007/s00167-020-06101-6

**Published:** 2020-06-17

**Authors:** Christian Ries, Christoph Kolja Boese, Nadine Ott, Jonas Doerner, Lars Peter Müller, Michael Hackl

**Affiliations:** 1 Department of Orthopaedics, University Medical Center Hamburg-Eppendorf, Martini Str. 52, 20246 Hamburg, Germany; 2 Department of Orthopaedic and Trauma Surgery, University Hospital of Cologne, Kerpener Str. 62, 50937 Cologne, Germany; 3 Institute of Diagnostic and Interventional Radiology, University Hospital Cologne, Kerpener Str. 62, 50937 Cologne, Germany

## Abstract

**Purpose:**

In cases of suspected rotational deformity of the lower limb, in particular in post-traumatic malalignment following closed nailing, there is a lack of adequate reference values. Available publications on leg rotation have either small sample sizes or do not include bilateral or whole leg rotation of healthy legs. This study aimed to determine side-specific reference values of lower limb rotation in a large healthy sample. This may be helpful in acute clinical settings as well as for medical expert opinion.

**Methods:**

226 consecutive bilateral lower limb computed tomography (CT) angiographies were screened. 105 patients (210 legs) were included (40 females, 65 males, mean age 67 ± 12 years). Bilateral axial femoral and tibial rotation alignment were independently measured and overall leg rotation was computed by two methods. Distributions, sex, and side differences were analyzed.

**Results:**

Two-sided paired *t* tests showed significant differences between right and left for all measurements. The left side showed a more pronounced mean anteversion in the femur of 2.2° (*p* = 0.002) and the right side higher mean external rotation in the tibia of 2.8° (*p* < 0.001). Overall leg rotation showed 5.1° more mean external rotation on the right side (*p* < 0.001) with both methods. Absolute side-to-side whole leg rotation difference was 9.5°. Absolute differences between both methods were 3.3°. The variance was high. 23 femora were retroverted, 1 tibia internally rotated, and 9 legs were overall internally rotated. No variables differed between female and male subjects except for femoral version (right *p* = 0.003 and left *p* = 0.002). Correlation coefficients were high (rho 0.550–0.934, all *p* < 0.001).

**Conclusion:**

There is a significant prevalence of side-to-side asymmetry in femoro-tibial torsion. Although side-to-side differences are not extraordinary, comparative axial femoro-tibial rotation alignment should always be interpreted with caution.

**Level of evidence:**

Diagnostic, retrospective cohort study, level III.

## Introduction

Rotational malalignment of the lower limb may result from congenital and acquired conditions including, but not limited to, osteotomies and post-traumatic deformity. In particular, open or closed reduction and intramedullary nailing of long bone fractures is a well-established and less invasive surgical procedure in the femur and tibia [1]. Closed nailing allows for stable fracture treatment and achieves indirect fracture healing in most cases. However, anatomical axial alignment reconstruction of femoral and tibial rotation is difficult, especially in comminuted fractures. Here, a lack of bony landmarks complicates adequate rotational orientation. Incorrect reconstruction may result in significant functional impairment. Axial-plane deformities of the lower limbs can affect the development of various knee disorders such as patella-femoral instability or knee and hip osteoarthritis [5].

In cases of suspected rotational deformity of the lower limb, tomography-based imaging analysis is the standard of care to determine the axes. The axial rotational alignment of the lower limb can be determined by four main axes of the two long bones: the femoral neck axis, the distal femoral condylar axis, and the proximal and distal tibial axes. The rotation of femur and tibia is calculated and summarized into the lower leg rotation. The contralateral healthy limb is usually used to determine the degree of abnormality of the affected bone by measuring side-to-side differences. However, individual side differences are not taken into account and may result in misjudgement. Rotational differences of up to 15°, compared to the healthy side, are considered to be acceptable after fracture reduction and internal fixation [8, 9, 14]. From a clinical point of view, external rotation is usually considered less relevant than internal rotation malalignment due to the decreased risk of stumbling. Increased internal rotation should, therefore, be avoided to ensure unrestricted walking.

Notably, there is a distinct lack of reference values from healthy subjects to interpret measurements. Available publications on leg rotation have either small sample sizes or do not include bilateral or whole leg rotation of healthy legs. The study hypothesis was that there is a significant side-to-side difference in intra-individual axial rotation alignment of the femur, tibia, and the whole lower limb in healthy subjects. The aim was to identify side differences and generate reliable reference values to improve an objective assessment of axial-plane deformities of the lower limbs in either post-traumatic or other conditions. It may be helpful in acute clinical settings as well as for medical expert opinion.

## Methods

In this retrospective study, all consecutive cases who underwent lower limb CT angiography between January 2017 and December 2019 in the Institute of Diagnostic and Interventional Radiology at University Hospital Cologne were identified using the hospital’s Picture Archiving and Communication System (PACS) (IMPAX EE, Agfa HealthCare GmbH, Germany).

**Fig. 1:**
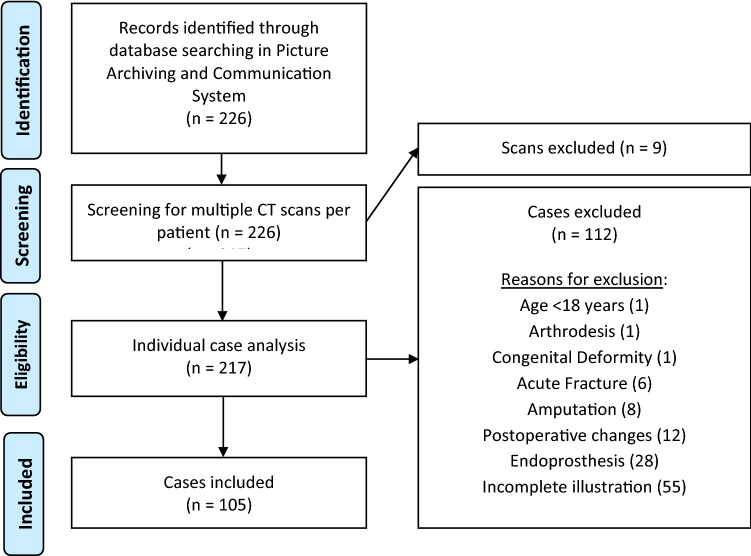
Flowchart of patient inclusion process

Cases with complete lower leg CT angiography depicting the complete femur and tibia with talus on both sides were eligible for inclusion. Patients who met at least one of the following criteria were excluded from the study: younger than 18 years of age, severe osteoarthritis of the hip and knee joint with joint deformity (Kellgren and Lawrence grade 4), endoprosthesis of the hip or knee joint, postoperative changes of the lower leg (e.g., after osteosynthesis), post-traumatic changes of the lower leg (e.g., acute fracture), bony abnormalities (e.g., tumors or severe deformities), amputation of the lower leg/thigh or incomplete illustration of bony landmarks in lower limb angiographies, and repeated CT scans (only one scan meeting the inclusion criteria was randomly used per patient). All other patients were included in the present study. 105 (210 legs) of 217 patients met the inclusion criteria and were enrolled in the study [40 females, 65 males, mean age 67 ± 12 years]. Patient selection is presented as a flowchart in Fig. 1.

The study was approved by the local ethics committee (ethics committee of the University Hospital of Cologne; approval number 19-1638) and followed the most recent version of the Declaration of Helsinki. Due to the retrospective design, no written patient consent was required.

### Radiological analysis and measurements

Lower limb CT angiographies were used for axial femoral and tibial rotation alignment analysis. Patients were kept stable in supine position with extended lower limbs during the standardized CT examination protocol. The lower limbs were not additionally fixed or strapped on the table. CT scans were performed using commercial CT systems (Brilliance iCT and iQON Spectral CT, Philips, The Netherlands) with constant reconstruction parameters. Sections of 1.5 mm thickness were reconstructed from raw data using a dedicated bone kernel.

Axial orientation was used to measure the radiographic parameters of femoral and tibial torsion. Measurements were acquired digitally using a commercially available picture archiving system (IMPAX EE, Agfa HealthCare GmbH, Germany). Measurements were performed independently by two experienced board certified orthopedic and trauma surgeons who are familiar with rotational analyses (CR, MH).

Femoral and tibial rotation alignment was measured according to Folinais et al. [5], previously described by Reikerås and Høiseth [16, 17] and validated by Liodakis et al. [14]. Overall leg rotation was computed by two methods.

**Fig. 2:**
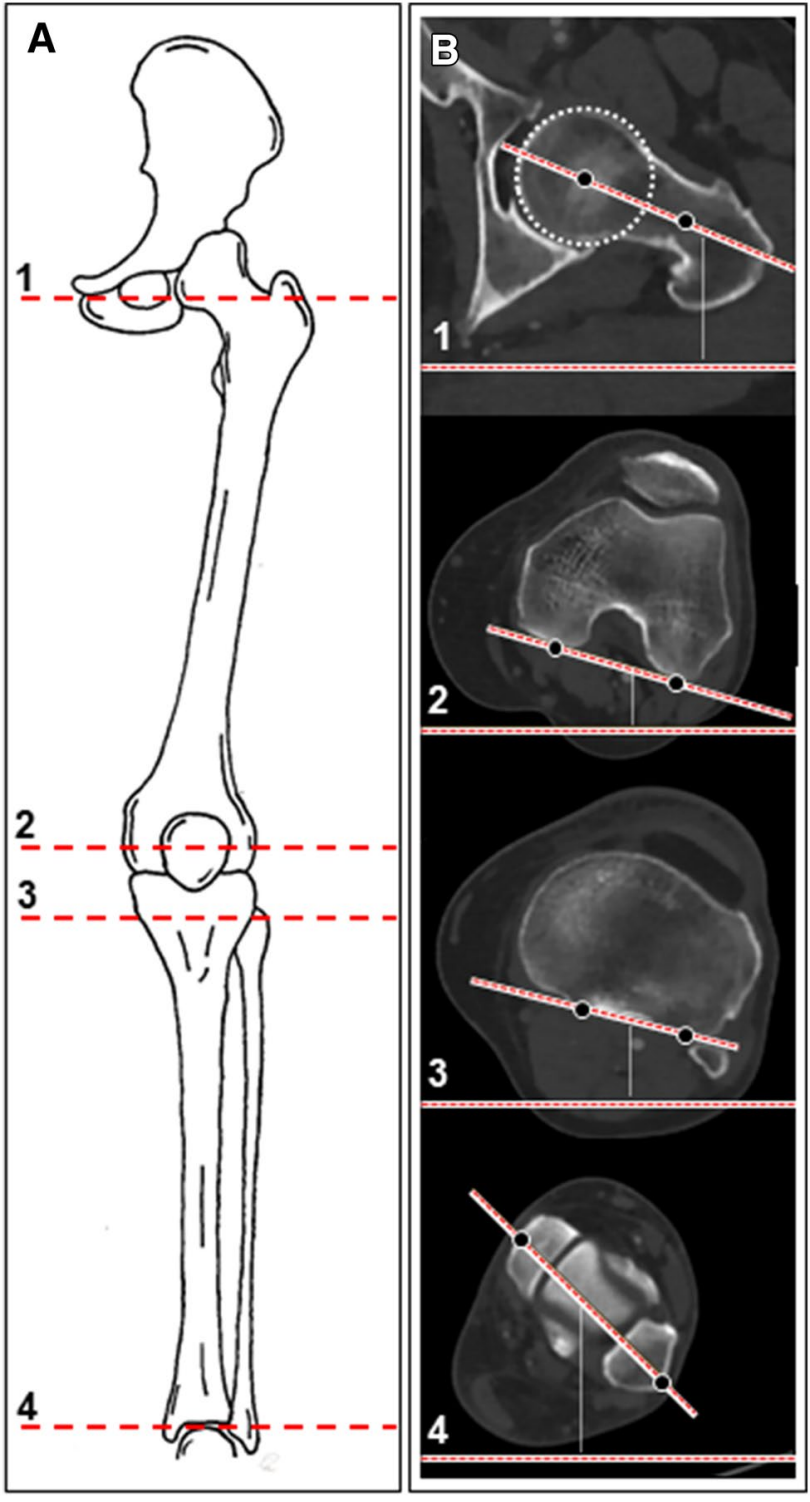
**a** Exemplary sketch of left lower limb coronal view. **b** Lower limb axial rotation alignment measurements. (1) Femoral neck axis. (2) Distal femoral condylar axis. (3) Proximal tibial axis. (4) Distal tibial axis (bimalleolar axis)

Four axes were measured, two in the femur and two in the lower leg (Fig. 2a, b): (1) femoral neck axis, (2) posterior condylar line (PCL) of the distal femur, (3) axis of the proximal tibia condyles, and (4) the bimalleolar axis.

Femoral version of the distal femur was measured by the angle formed between the line that intersects the femoral neck and the line indicating the posterior condylar line (PCL) of the distal femur. Femoral neck axis was defined according to Hernandez et al. [7] by the line between the center of the femoral head and the isthmus of the neck in a CT cut where the femoral head, isthmus of the femoral neck, and the superior border of the greater trochanter are evident. Positive values represent femoral anteversion and negative values represent retroversion of the femoral neck in relation to the PCL. Femoral anteversion equals femoral internal rotation of the PCL in relation to the femoral neck.

Tibial torsion was measured by the angle between a line connecting the posterior aspects of the proximal tibial condyles and the bimalleolar axis. The line connecting the posterior aspects of the proximal tibial condyles was set at the level of the apex of the fibula. The bimalleolar axis is drawn in a cut just below the tibial pilon’s articular surface with the medial and lateral malleoli and talar dome evident between the centers of the dense surfaces of the malleoli [6, 14]. External rotation of the tibia is represented by negative values. Positive values indicated internal rotation of distal tibia in relation to the baseline of the proximal posterior tibia plateau.

Leg rotation is reported as method 1: overall axial lower limb rotation was determined by relative rotation of the femoral neck axis to bimalleolar axis. Negative values represented a relative external rotation of the distal tibia to the proximal femur. This leg rotation includes potential rotational elements due to knee laxity (leg rotation with knee). An alternative lower leg rotation was reported as method 2. It is the difference of separate femur rotation/version and tibial rotation. It eliminates potential leg rotation due to knee laxity (leg rotation w/o knee). Overall leg rotation is defined as external rotation for negative and internal rotation for positive values.

### Statistical analysis

For descriptive analysis, mean values and standard deviations (SD) of the measured variables are reported. One decimal place is reported for all variables. Variables were tested against normality using the Shapiro–Wilk test. All variables except for leg rotation (both methods left leg and difference of both methods) were normally distributed. Due to large sample sizes, parametric tests were used for all variables. Significance levels for alpha error were set at 0.05. Two-sided independent paired t tests were performed for sex and side differences. Differences of variables are reported as relative differences (positive and negative values) as well as absolute values (only positive values). For reliability analysis, intraclass correlation coefficients (ICC) were calculated using a two-way mixed model with absolute agreement.

IBM SPSS Statistics 25 for Mac (Statistical Package for the Social Sciences version 25, IBM Corporation, Armonk, NY) and Microsoft Excel for Mac version 15.41 (Microsoft Corporation, Redmond, WA) were used.

## Results

**Fig. 3:**
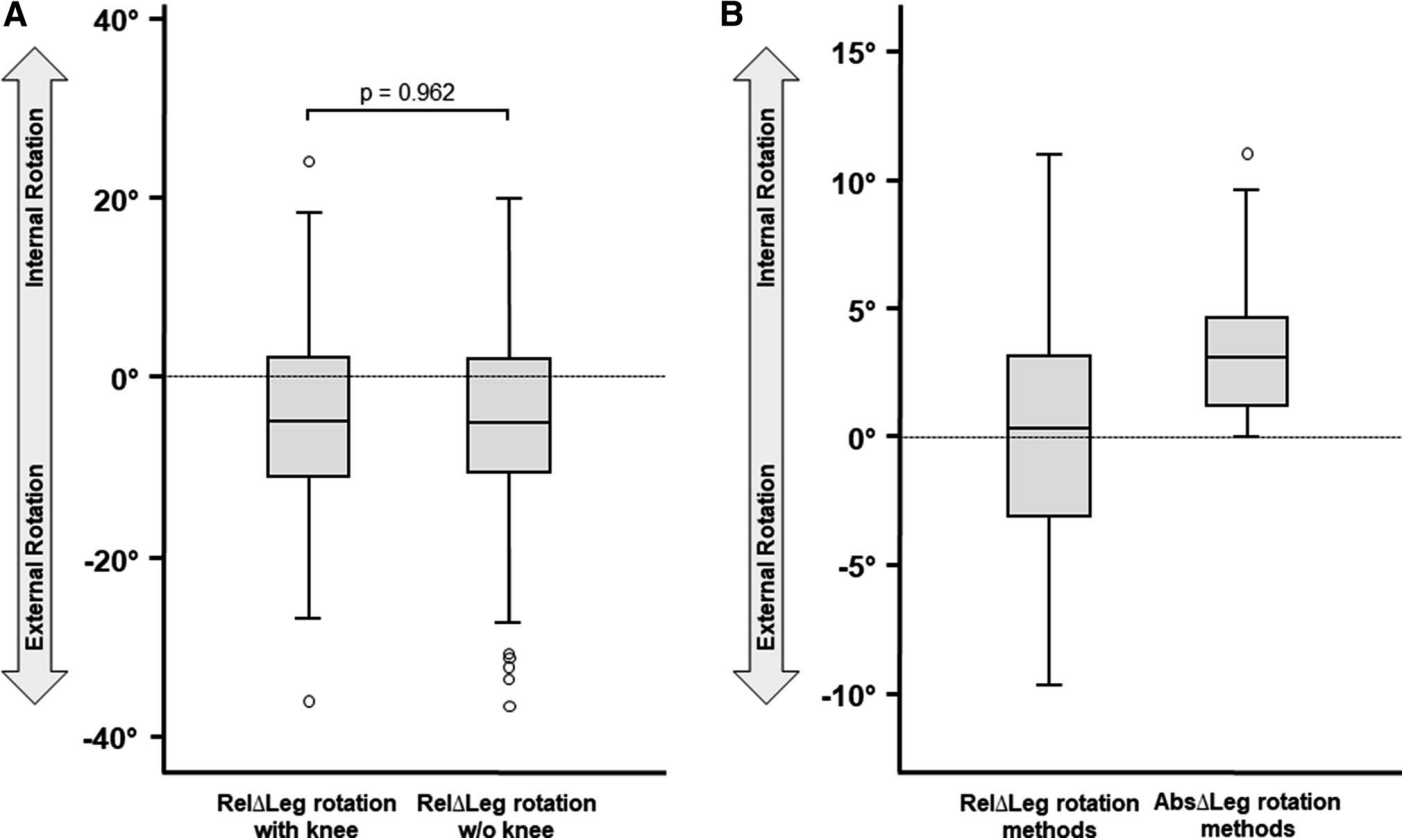
Measurement results of methods 1 and 2 for whole leg rotation depicted by Box-Plot diagrams

Descriptive analysis of side-specific rotation analysis is provided in Table 1. Two-sided paired t tests showed significant differences between right and left for all measurements. The left side showed a more pronounced mean anteversion in the femur of 2.2° (*p* = 0.002) and the right side more mean external rotation in the tibia of 2.8° (*p* < 0.001). Overall leg rotation showed 5.1° more mean external rotation on the right side (*p* < 0.001) with both methods (Fig. 3a). The absolute side-to-side whole leg rotation difference was 9.5°. Absolute differences between both methods were 3.3° (Fig. 3b). The variance was high. The number of anteverted/retroverted femora and external/internal rotation of tibiae as well as whole legs is reported in Table 2.

**Table 1:** Baseline characteristics of measurements shown for all subjects

	Side		Side-to-side		*p* value of RelΔ
	Right mean ° (SD)	Left mean ° (SD)	RelΔ (right–left) mean ° (SD)	Abs Δ (right–left) mean ° (SD)	
Femur version	+ 10.1 (9.3)	+ 12.4 (8.9)	− 2.2 (7.4)	6.0 (4.7)	*0.002*
Tibia rotation	− 32.7 (9.9)	− 29.9 (9.6)	− 2.8 (6.9)	5.7 (4.8)	< *0.001*
Leg rotation with knee (method 1)	− 24.7 (12.0)	− 19.6 (11.3)	− 5.1 (11.1)	9.5 (7.6)	< *0.001*
Leg rotation w/o knee (method 2)	− 22.6 (13.1)	− 17.6 (11.9)	− 5.1 (11.4)	9.5 (8.1)	< *0.001*
ΔLeg rotation method with knee vs. w/o knee	–	–	0.0 (4.1)	3.3 (2.4)	0.962

Values are given as means and standard deviations (SD)

RelΔ relative difference, AbsΔ absolute difference

Significance levels for alpha error were set at 0.05

**Table 2:** Frequencies of rotation patterns by side

	Side	
	Right (*n*)	Left (*n*)
Femur/hip anteversion	92	95
Femur/hip retroversion	13	10
Tibia external rotation	105	104
Tibia internal rotation	0	1
Whole leg (w/o knee) external rotation	101	100
Whole leg (w/o knee) internal rotation	4	5

Sex-specific rotation parameters are provided in Table 3. No variables differed between female and male subjects except for femoral version (right  *p* = 0.003 and left *p* = 0.002).

**Table 3:** Baseline descriptive statistics separately shown for male and female subjects

	Sex				*p* value
	Female (*n* = 40)		Male (*n* = 65)		
	Mean [°]	SD [°]	Mean [°]	SD [°]	
Age (years)	65.1	12.4	68.3	12.4	0.204
Femur version (right)	+ 13.5	10.6	+ 8.1	7.9	*0.003*
Femur version (left)	+ 15.7	10.1	+ 10.3	7.4	*0.002*
RelΔFemur version (right–left)	− 2.2	7.6	− 2.3	7.3	0.978
AbsΔFemur version (right–left)	6.0	5.1	6.1	4.6	0.948
Tibia rotation (right)	− 33.7	10.3	− 32.1	9.7	0.416
Tibia rotation (left)	− 31.5	11.0	− 28.9	8.5	0.169
RelΔTibia rotation (right–left)	− 2.2	7.9	− 3.2	6.2	0.463
AbsΔTibia rotation (right–left)	6.6	4.7	5.1	4.8	0.116
Leg rotation with knee (right)	− 22.9	12.7	− 25.8	11.6	0.220
Leg rotation with knee (left)	− 19.4	14.3	− 19.8	9.1	0.853
RelΔLeg rotation with knee (right–left)	− 3.5	12.4	− 6.0	10.1	0.253
AbsΔLeg rotation with knee (right–left)	10.3	7.6	9.0	7.6	0.371
Leg rotation w/o knee (right)	–20.3	14.2	–24.0	12.3	0.153
Leg rotation w/o knee (left)	–15.9	14.8	–18.6	9.7	0.260
RelΔLeg rotation w/o knee (right–left)	− 4.4	13.2	− 5.5	10.3	0.647
AbsΔLeg rotation w/o knee (right–left)	10.9	8.5	8.6	7.8	0.177
RelΔLeg rotation methods	+ 0.9	4.2	− 0.6	4.0	0.071
AbsΔLeg rotation methods	3.4	2.7	3.3	2.2	0.886

Values are given as means and standard deviations (SD)

*Rel*Δ relative difference, *Abs*Δ absolute difference

Significance levels for alpha error were set at 0.05

ICCs were between 0.986 and 0.998 (*p* < 0.001) for all measurements.

Pearson’s correlation coefficients were high between right and left femur version (rho = 0.674, *p* < 0.001), right and left tibia rotation (rho = 0.751, *p* < 0.001) as well as right and left leg rotation with both methods (Method 1 with knee: rho = 0.550, *p* < 0.001; Method 2 w/o knee: rho = 0.584, *p* < 0.001). Correlations between the methods on either side (with vs. w/o knee) were high for right legs (rho = 0.908, *p* < 0.001) and left legs (rho = 0.893, *p* < 0.001). Subsequently, the whole leg rotation differences (right–left) of both methods correlated highly (rho = 0.934, *p* < 0.001).

## Discussion

The most important findings of the present study were significant differences between total lower limb rotation of both sides as well as rotation of femur and tibia alone in healthy subjects. This finding is of utmost importance to adequately assess leg rotation in subjects with assumed axial-plane deformities of the lower limbs in either post-traumatic or other conditions.

Besides correction for deformities, fractures of long bones require restoration of correct rotational limb alignment [9]. Comminuted fractures are associated with a loss of anatomical landmarks to identify the native rotation. Reconstruction of the pre-traumatic alignment of the affected bones is a main goal. Here, the healthy side is usually used as reference [19]. The incidence of rotational malalignment following closed nailing of femoral and tibial diaphyseal fractures admittedly reaches up to 22% and 28%, respectively [9, 14, 15]. Rotational differences of more than 15°, compared to the healthy side, are considered true deformities and corrective procedures may be indicated [8, 9, 12, 14, 19]. However, patients may complain about lesser deformities and ask for revision surgery.

Nevertheless**,** there is a lack of reliable reference values based on bilateral healthy legs to guide treatment pathways and methods to assess rotation. CT rotation analysis of both legs is the standard of care for rotation analysis [13], but depends on the level of selected CT slices. Inaccuracies in the identification landmarks can lead to major measurement differences [3–5].

Liodakis et al. [14] validated several CT methods and stated that the most reliable methods are the Hernandez [7] and bimalleolar methods [10, 11, 17] for measuring femoral and tibial torsion, respectively. Folinais et al. [5] observed a femoral torsion of 13.7° ± 9.4° and a tibial torsion of 30.3° ± 9.6° in 43 lower limbs by CT and EOS studies in 30 patients. However, side-to-side differences and overall leg rotation were not analyzed in detail. The present study found comparable mean femoral torsion of 11.3° ± 9.1° and mean tibial torsion of 31.3° ± 9.8° by axial rotation alignment measurements according to Folinais et al. [5]. Chang et al. [2] reported femoral anteversion likewise in reference to the PCL with 13.2° ± 8.3°. However, external tibial rotation (27.2° ± 6.8°) was measured by connecting the most prominent points of medial and lateral malleolus with the fibula articulating in the incisura fibularis. Therefore, direct comparisons are limited.

Previously, studies on limb rotation were mostly limited by inclusion of traumatized or deformed limbs. Strecker et al. [18] evaluated the torsion of the lower limb in 355 adult patients using the Ulm method [19]. However, only 48 legs (24 per side) were available for measurement of a healthy femur and tibia in both sides. Median differences of bilateral torsion were 4° in 172 paired femora, 4.5° in 176 paired tibiae, and 5° in 24 paired whole legs. Waidlich et al. [19] analysed femoral and tibial torsion in 50 patients using postoperative CT scans after closed nailing of diaphyseal shaft fractures. The contralateral healthy side and the non-injured level of the lower limb were used to determine side-to-side differences in a population including children and young adults. Side-to-side mean differences of 4.3° ± 2.3° in the femur and 6.1° ± 4.5° in the tibia were reported in 19 cases [19]. These results are in line with results from the present study with absolute mean side-to-side differences of 6.0° ± 4.7° in femoral and 5.7° ± 4.8° in tibial torsion. Waidlich et al. [19] concluded that only angles greater than 9° in the femur and 15° in the tibia should be regarded as abnormal.

Additional studies supported the theory of intra-individual side differences in leg rotation. A high prevalence of side-to-side asymmetry of about 6° for femoral torsion and 4° for tibial torsion was described in the previous studies [16–18]. In 1989, Reikerås and Høiseth [17] measured the torsion of the lower leg in 50 adults by computed tomography (proximal reference line: tangent to the dorsal aspect of the femoral condyles; distal reference line: between centers of the medial and lateral malleoli). The authors reported mean leg external rotation on the right side of 39.5° ± 7.5° and 38.5° ± 10.5° on the left side. However, calculation of whole leg rotation was not performed using independent analysis of femoral and tibial axial torsion. Thus, the knee remains a potential bias due to soft-tissue conditions that can affect the dynamic alignment of the lower limb. In the present study, two methods were reported to additionally identify potential influences on the knee joint. Calculation of whole leg rotation revealed relative mean differences of 5.1° (9.5° absolute difference) with both methods. Absolute differences for both lower limb rotation measurements were 3.3°, showing a potentially clinically relevant difference between the methods. Therefore, the authors prefer the separate measurement of femur and tibia for the assessment of leg rotation (method 2).

There are limitations to the present study. First, the cohort of 105 patients undergoing CT angiography does not represent the general population. The majority of included patients were elderly with an average age of 67 years. Age-related changes in bone morphology, therefore, cannot be ruled out. Due to ethical considerations, this group was considered the best choice for whole leg CT scans in otherwise healthy subjects. Second, the interpretation of rotation analysis requires standardized and reliable methods. The identification of reliable landmarks was performed following a strict protocol based on validated methods. Still, different methods were reported in the literature limiting direct comparison between studies [14]. Third, CT scans are static and, therefore, soft-tissue conditions that can affect the dynamic alignment of the lower limb are not reflected by method 1. Another method was, therefore, added to calculate whole leg rotation with independent analysis of the femur and tibia, eliminating the knee as potential bias. The differences between both methods were non-significant. Yet, an absolute difference of 3.3° was observed. It may be considered to recommend reporting both methods in future publications.

## Conclusion

In conclusion, significant side-to-side differences of bilateral lower limb axial femoro-tibial rotation alignment were observed. Although side-to-side differences are not extraordinary, comparative axial femoro-tibial rotation alignment should always be interpreted with caution in decision-finding for diagnosis and therapy of congenital and acquired rotational leg deformities. These findings may be helpful to guide medical expert opinion.
